# The importance of examining early maladaptive schemas in the diagnosis and treatment of obsessive-compulsive disorder

**DOI:** 10.3389/fpsyt.2024.1360127

**Published:** 2024-05-10

**Authors:** Katalin Csigó, Ákos Münnich, Judit Molnár

**Affiliations:** ^1^Institute of Psychology, Pázmány Péter Catholic University, Budapest, Hungary; ^2^Psychotherapy Center, Nyírő Gyula National Institute of Psychiatry and Addictions, Budapest, Hungary; ^3^Department of Behavioural Sciences, Faculty of Medicine, University of Debrecen, Debrecen, Hungary

**Keywords:** OCD, maladaptive schemas, comorbid anxiety and depression, dimensional view of OCD, schema theories

## Abstract

**Introduction:**

The aim of the study was twofolded: to identify the early maladaptive schemas characteristic of obsessive-compulsive disorder in a Hungarian sample and, to examine the presence and severity of comorbid anxiety and depressive symptoms in the light of early maladaptive schemas.

**Methods:**

112 participants (58 men and 54 women) diagnosed with OCD were involved in the study. The questionnaire package consisted of the Yale-Brown Obsessive Compulsive Scale (Y-BOCS), the State-Trait Anxiety Inventory (STAI), the Beck Depression Inventory (BDI), the Penn State Worry Questionnaire (PSWQ) and the Schema Questionnaire (SQ).

**Results:**

We identified five early maladaptive schemas with a direct effect on the manifestation of obsessive-compulsive symptoms: Mistrust-Abuse, Inferiority/Shame, Dependence/Incompetence, Insufficient Self-Control/Self-Discipline and Entitlement/Grandiosity (reversed effect). Based on the severity of the early maladaptive schemas, three significantly different groups could be identified in our sample: patients with mild, moderate and high schema-values. Among the groups significant differences can be found in the appearance and severity of compulsive symptoms, as well as in the presence of anxiety and depressive symptoms. But contrary to our expectations, not the severity, but the *number*of the early maladaptive schemas showed a stronger correlation with the symptom variables. An additional result of our study derives from canonical correlation, addressing the relationship among early maladaptive schemas, OCD symptoms, anxiety and depressive symptoms from a new perspective. The results highlight that OCD is only one and not the most serious consequence of personality damage, indicated by early maladaptive schemas.

**Discussion:**

The results of our study suggest that obsessive-compulsive disorder can be divided into several subgroups, which can be separated in terms of symptom severity, comorbid psychiatric symptoms and personality impairment patterns. The relationship between OCD symptom severity and personality impairment seems to be not directly proportional. Our results strengthen the new dimensional view of OCD, which can determine the selection of the appropriate therapeutic treatment method beyond the diagnostic process.

## Introduction

Obsessive-compulsive disorder (OCD) is a clinical phenomenon consisting of compulsive thoughts and compulsive actions. The symptoms of the disease are extremely diverse, with heterogeneous manifestations. The course of OCD is sometimes chronic, severe, and often therapy-resistant (1–3). Obsessive-compulsive disorder is the fourth most common psychiatric disease, with a prevalence of 1-3% (4). The rate of subclinical forms is much higher, between 10 and 15% (5), but according to other studies, this proportion can reach 40% among the adult population (6). The diagnostic and differential diagnostic process of OCD is based on clinical exploration and self-completed questionnaires (e.g. Y-BOCS).

The treatment protocol for OCD recommends a combination of CBT and pharmacotherapy as the leading method (7, 8), since, despite proper therapy choices, research data shows that nearly 25% of patients do not respond adequately to treatment (9). According to other data, this number can be much higher, at around 50% (10). In these cases symptoms persist, and the recommended treatment does not cause remission.

These findings raise important questions: is the diagnosis of OCD defined in an appropriate way? Are comorbid diagnoses identified adequately? Are the characteristic symptoms of the patient well understood, or should our approach be changed (see, for example, the dimensional approach)? Overall, might it be necessary to look at obsessive-compulsive disorder from a new perspective (11)?

Non-responder OCD patients are characterized by early onset of the disease, early childhood traumatization and emotional deprivation, as well as comorbid personality disorder. In this context, Sookman (12) already proposed a complex approach to OCD, including the early attachment experiences of the patients, developmental theory, the structural dimension, emotional focus and schemas. The complex examination of these factors can be conducted by using a new approach in the study of OCD, i.e. the empirical and theoretical frame of maladaptive schemas.

The concept of early maladaptive schemas was described by Jeffrey E. Young, who conceptualizes mental problems with the presence and persistence of these schemas. Early maladaptive schemas can be defined as mental patterns consisting of pervasive memories, feelings, and cognitions related to ourselves and our relationships with others (13).

Empirical findings regarding the presence of early maladaptive schemas in OCD are contradictory, so it is not clear which schema modes could be used to describe the disorder (14).

After reviewing research conducted with the Young Schema Questionnaire, the studies can be divided into four groups.

The first group of studies make efforts to identify the characteristic schema profile of obsessive-compulsive disorder. Results suggest higher values on Social isolation/Alienation, Vulnerability to Harm; Negativism-Pessimism (15); on Inferiority-Shame and Social Isolation/Alienation (16); an increased value on the Vulnerability to Harm schema (17); and higher values on Emotional Deprivation, Mistrust/Abuse and Defectiveness/Shame schemas (23).

Another group of studies aims to describe the schemas characteristic in OCD by comparing obsessive-compulsive patients with normal controls. Kim et al. (18) compared schema modes of OCD patients with normal controls and found that OCD patients show higher values on the Inferiority/Shame, Social Isolation/Alienation and Failure to Achieve schemas. Another novel aspect of their study was that the activation of the schemas was compared with the OCD symptom dimensions, and they found that the Vulnerability/Endangerment and Enmeshment/Undeveloped self schema showed a connection with the sexual/religious compulsion symptom dimension. Compared to normal controls in another study, Enmeshment/Undeveloped self, Failure to achieve, Negativism-pessimism, Vulnerability to harm, Emotional deprivation, Social isolation, Defectiveness-shame, Approval seeking, Insufficient self-control, Self-sacrifice and Punitiveness schemas were described as having high values (19).

The third group of studies attempted to determine the schema profile specific to OCD compared to other psychiatric diseases.

Comparing obsessive-compulsive patients with trichotillomaniac patients it was found that Emotional deprivation, Social isolation/Alienation, Mistrust-abuse, Defectiveness-shame, Subjugation and Emotional inhibition schemas show significantly higher values in obsessive-compulsive patients (20). In an Iranian sample, a high value of the Mistrust/Abuse schema was found to be characteristic for OCD, and it also proved to be related to a high risk of suicide (21).

The last group of tests tries to measure and verify the effectiveness of the treatment in the light of maladaptive schemas.

In Wilhelm (22) study, as a result of CBT, a measurable change appeared in the Dependency/Incompetence schema, preceding adaptive changes in behavior. Furthermore, Haaland et al (24) found that a high Abandonment/Instability schema value measured before treatment was associated with a worse treatment outcome, while a high Self-Sacrifice schema indicated a good treatment outcome. According to Gross et al. (25), obsessive-compulsive disorder can be described as a special functioning of the personality, where compulsive symptoms are the results of overcompensation, manifesting themselves in the Perfectionist/Overcontrolling mode or in the Alienated/Protective mode. In this conceptual framework, these two characteristic schema modes are related to the Vulnerable Child Mode and the Demanding Parent Mode. Alienation makes the therapeutic relationship difficult, while overcontrolling hinders participation in exposure-response prevention therapy (25).

Systematic reviews prove that schema therapy is a promising treatment approach in the therapy of OCD patients (26, 27), but at the same time the case study data available is both sporadic and limited in number. The schema therapy approach can be particularly useful for those who do not respond properly to CBT techniques, are characterized by a chronic lifestyle, have a history of trauma or suffer from a comorbid personality disorder (25).

Research related to schemas and schema modes in obsessive-compulsive disorder is quite contradictory, which can be caused by several factors. On the one hand, the measurement of compulsive symptoms in the studies is problematic – the symptoms are measured with a self-administered Y-BOCS test and/or the Y-BOCS test filled out by the clinician, which can cause significant differences in the results. On the other hand, results can vary, depending on whether the studies control for comorbid psychiatric diseases. The methods used to analyze the Schema Test also vary in different research, in terms of where the high schema value is defined, and which value is considered elevated. It is also an open question whether the schemas measured in the studies are OCD-specific, or their presence is rather caused by other factors (e.g. comorbid personality disorder, OCD as a personality dysfunction, OCD as a chronic disease course). Examining schemas in obsessive-compulsive disorder is therefore particularly important both from a psychodiagnostics point of view and for treatment planning.

## The aim of the study

Our research was based on two goals. Our first aim was to identify the early maladaptive schemas characteristic of obsessive-compulsive disorder, as they have not yet been assessed in a Hungarian sample. The second goal of the study was to examine the presence and severity of comorbid anxiety and depressive symptoms in the light of early maladaptive schemas.

## Method

### Participants and procedures

The patients in the study participated in the OCD-specific psychotherapy treatment program of the OMIII (National Institute of Mental Health, Neurology and Neurosurgery, Budapest). The inclusion criterion was the diagnosis of obsessive-compulsive disorder based on the DSM-5 (DSM-5 28). The diagnosis was established by both a psychiatrist and a clinical psychologist. Exclusion criteria included a diagnosis of psychosis, autism, and mental retardation. The patients participating in the study were informed about the anonymous handling of their questionnaire data and their use as research data. The study was approved by the Medical Research Council.

The entire sample consists of 112 people, 58 of whom are men, and 54 women. The average age of the sample is 31.4 years. **Table 1**

**Table 1:** Demographic data of the sample.

Education							
		grade school	university	high school	college	vocational school	Total
Gender	Male	1	26	22	4	1	54
	Female	0	16	34	7	1	58
Total		1	42	56	11	2	112

### Measures

The Yale-Brown Obsessive Compulsive Scale (Y-BOCS) (29; Hungarian adaptation Arató M.) is a self-administered symptom assessment scale most often used in obsessive-compulsive disorder. The scale consists of two parts. In the first part the patient indicates whether the given symptom has occurred in the present or in the past for each of the 70 listed symptoms. In the second part, the clinician scores the severity of the obsessions and compulsions on a 10-point scale. In the study, we used the patients’ self-scoring questionnaire.

The State-Trait Anxiety Inventory (STAI; 30; Hungarian adaptation 31) is a widely used 40-item self-completion questionnaire developed to measure the intensity of anxiety. There are two versions, the current state anxiety (STAI-S) and the anxiety as a personality trait (STAI-T) scales.

The Beck Depression Inventory (BDI, 32; Hungarian adaptation 33) is one of the most frequently used questionnaires to determine and monitor the severity of depression.

The Penn State Worry Questionnaire (34, 35) is a 16-item self-administered questionnaire most often used to measure pathological worry.

The Schema Questionnaire (13; Hungarian adaptation 36) is a tool for measuring early maladaptive schemas responsible for the development and persistence of pathological personality traits. The Hungarian version of the questionnaire contains 19 maladaptive schemas, which can be classified into five higher schema ranges. The internal consistency of the Hungarian version of the questionnaire is very high (Cronbach’s alpha=0.86-0.95). Its factor structure is consistent with the results obtained on international samples, and its validity has been confirmed too (36).

### Statistical analysis

As the first step of the statistical analysis, we filtered the data. Due to improper filling out of the questionnaires or missing answers we had 97 participants who completed both the Y-BOCS and the Schema Questionnaire. So in analyzing data relevant for the first objective of the study we worked on a sample of 97 participants. In the examination of our second goal we analyzed answers from those who filled out all questionnaires properly, i.e. the responses of 78 people. **Table 2**

**Table 2:** Descriptive statistics of Y-BOCS, STAI-S, STAI-T, BDI and PSWQ questionnaires.

	N	Minimum	Maximum	Mean	Std. Deviation
Y-BOCS	97	3	98	32.67	21.637
STAI-S	110	23	78	51.30	12.877
STAI-T	108	29	78	55.38	11.122
BDI	90	0	40	16.06	9.208
PSWQ	110	30	80	61.45	10.998
Valid N (listwise)	78				

In order to calculate the schema values, the answers indicated by 3, 4, 5 or 6 per person and schema were taken into account. We divided the number obtained in this way by the number of possible choices and multiplied it by 100, thus obtaining a percentage value. The value is 100% if the examinee gave all questions a value of 3 or more.

Based on the values of the 19 early maladaptive schemas, we classified the respondents into three clusters (characterized by low, medium and high schema values) using K-means cluster analysis.

In the next step we examined the effect of specific early maladaptive schemas on the value of Y-BOCS using multiple linear regression analysis, where the target variable was Y-BOCS and the predictors were schema variables; then we checked the deviation of the Y-BOCS according to the clusters using variance analysis.

Finally, Pearson’s correlations between the Y-BOCS and the STAI-S, STAI-T, BDI and PSWQ variables were calculated.

## Results

The mean values (percentages) of Y-BOCS and early maladaptive schemas in the sample can be seen on **Table 3**.

**Table 3:** Mean values and standard deviation of Y-BOCS and specific early maladaptive schemas (percentage) for the entire sample.

	Mean	Std. Deviation	N
Y-BOCS	32.67	21.63	97
Ed	38.83	36.78	97
Ab	43.52	29.22	97
Ma	41.96	26.12	97
Si	48.76	32.34	97
Ds	33.26	29.03	97
Su	31.04	29.56	97
Fa	39.17	35.06	97
Di	45.77	30.45	97
Vh	48.82	28.03	97
Em	37.95	29.92	97
Et	44.14	26.11	97
Is	50.44	26.57	97
Sb	45.05	32.56	97
Ss	57.36	27.23	97
As	55.74	29.31	97
Np	64.76	25.85	97
Ei	43.75	27.81	97
Us	60.88	25.5	97
Pu	54.41	25.28	97

**Figure 1:**
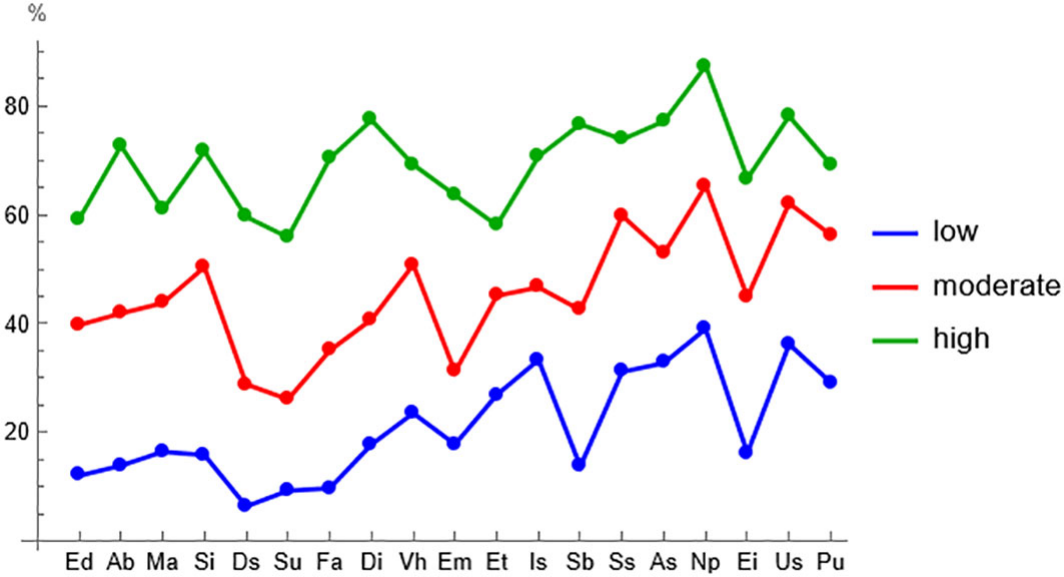
Clusters of early maladaptive schemas characterized by low, medium and high values.

The cluster profiles characterized by low, medium and high maladaptive schema values can be seen in **Figure 1**.

Linear regression analysis showed a significant effect on the Y-BOCS value in the case of the following early maladaptive schemas: Mistrust-Abuse, Inferiority/Shame, Dependence/Incompetence, Entitlement/Grandiosity and Insufficient Self-Control/Self-Discipline. In the case of the specific early maladaptive schemas of Mistrust-Abuse, Inferiority/Shame, Dependence/Incompetence and Insufficient Self-Control/Self-Discipline the effect is direct, while in the case of the Entitlement/Grandiosity schema it is reversed.

The early maladaptive schemas above belong to three major schema domains: Disconnection and Rejection (Ma, Ds), Impaired Autonomy and Performance (Di), and Impaired Limits (Et, Is) (**Table 4**).

**Table 4:** Results of linear regression, where the target variable is the value of Y-BOCS and the predictors are the values of early maladaptive schemas.

Model		Unstandardized Coefficients		Standardized Coefficients	t	Sig.
		B	Std. Error	Beta		
1	(Constant)	16.030	6.660		2.407	.018
	Ed	.001	.074	.002	.015	.988
	Ab	-.081	.119	-.110	-.680	.499
	Ma	.288	.116	.347	2.479	.015
	Si	-.151	.107	-.226	-1.412	.162
	Ds	.249	.114	.334	2.180	.032
	Su	-.044	.116	-.060	-.381	.704
	Fa	-.138	.092	-.223	-1.502	.137
	Di	.235	.117	.330	2.009	.048
	Vh	.125	.098	.162	1.279	.205
	Em	.029	.090	.041	.327	.745
	Et	-.313	.120	-.378	-2.603	.011
	Is	.248	.108	.304	2.287	.025
	Sb	-.131	.094	-.196	-1.389	.169
	Ss	-.104	.108	-.131	-.968	.336
	As	-.123	.100	-.166	-1.233	.221
	Np	.059	.113	.071	.522	.603
	Ei	.128	.098	.164	1.310	.194
	Us	.058	.101	.068	.572	.569
	Pu	.056	.102	.065	.546	.586

According to the results of Pearson’s correlation, all variables (STAI-S, STAI-T, BDI, PSWQ, Y_BOCS) correlate significantly. But in the light of our research goals, we focused only on the variables correlating with Y-BOCS. A moderately strong, significant relationship was found between YBOCS and STAI-T, while there were significant but weaker relationships between YBOCS and STAI-S, Y-BOCS and BDI, and PSWQ and Y-BOCS. **Table 5**

**Table 5:** The results of the Pearson correlation.

		Y-BOCS	STAI-S	STAI-T	BDI	PSWQ
Y-BOCS	Pearson Correlation	1	.282**	.411**	.296**	.311**
	Sig. (2-tailed)		.005	<.001	.009	.002
	N	97	97	96	78	97
STAI-S	Pearson Correlation	.282**	1	.582**	.517**	.444**
	Sig. (2-tailed)	.005		<.001	<.001	<.001
	N	97	110	108	90	110
STAI-T	Pearson Correlation	.411**	.582**	1	.690**	.637**
	Sig. (2-tailed)	<.001	<.001		<.001	<.001
	N	96	108	108	89	108
BDI	Pearson Correlation	.296**	.517**	.690**	1	.438**
	Sig. (2-tailed)	.009	<.001	<.001		<.001
	N	78	90	89	90	90
PSWQ	Pearson Correlation	.311**	.444**	.637**	.438**	1
	Sig. (2-tailed)	.002	<.001	<.001	<.001	
	N	97	110	108	90	110

** = strong correlation.

In the next step we examined the relationship between obsessive-compulsive symptoms, comorbid anxiety and depressive symptoms and the severity of maladaptive schemas (Cluster 1, 2 and 3). According to the results of the variance analysis, significant differences can be found in the groups characterized by low, medium and high schema values along with the total values of the STAI-S, PSWQ and BDI scales. For all variables examined, the highest mean scores were found not in group 3, as we had expected, but in group 2. Group 2 can be characterized by moderately severe schemas, but with the largest *number* of schemas (sum of schemes) **Table 6**.

**Table 6:** The results of the variance analysis.

		N	Mean	Std. Deviation	Std. Error	Minimum	Maximum
Y-BOCS	1	46	29.11	18.703	2.758	4	78
	2	28	43.11	22.872	4.322	12	90
	3	23	27.09	22.080	4.604	3	98
	Total	97	32.67	21.637	2.197	3	98
STAI-S	1	51	50.78	11.903	1.667	26	74
	2	35	56.69	13.139	2.221	23	78
	3	24	44.54	11.413	2.330	25	63
	Total	110	51.30	12.877	1.228	23	78
STAI-T	1	51	54.39	9.743	1.364	35	74
	2	33	63.73	8.921	1.553	45	78
	3	24	46.00	8.027	1.639	29	58
	Total	108	55.38	11.122	1.070	29	78
BDI	1	43	15.16	9.108	1.389	0	35
	2	27	21.19	9.195	1.770	7	40
	3	20	11.05	5.708	1.276	0	23
	Total	90	16.06	9.208	.971	0	40
PSWQ	1	51	59.80	11.128	1.558	30	80
	2	35	67.89	9.132	1.544	48	80
	3	24	55.58	8.707	1.777	35	75
	Total	110	61.45	10.998	1.049	30	80
Schema_total	1	52	112.92	15.856	2.199	83	143
	2	36	170.39	20.533	3.422	129	215
	3	24	53.38	18.812	3.840	24	83
	Total	112	118.63	46.115	4.357	24	215

In the final step we examined structural relationships among all variables by canonical correlation.

When the variables of an observation are partitioned into two psychological contexts, canonical-correlation analysis (CCA) can be used to explore possible structural relationships between the contexts. In our case context1 contains the variables of the comorbid symptoms of OCD (YBOCS, STAI-S, STAI-T, BDI, PSWQ), while context2 consists of the early maladaptive schemas (Ed, Ab, Ma, Si, Ds, Su, Fa, Di, Vh, Em, Et, Is, Sb, Ss, As, Np, Ei, Us, Pu).

**Figure 2:**
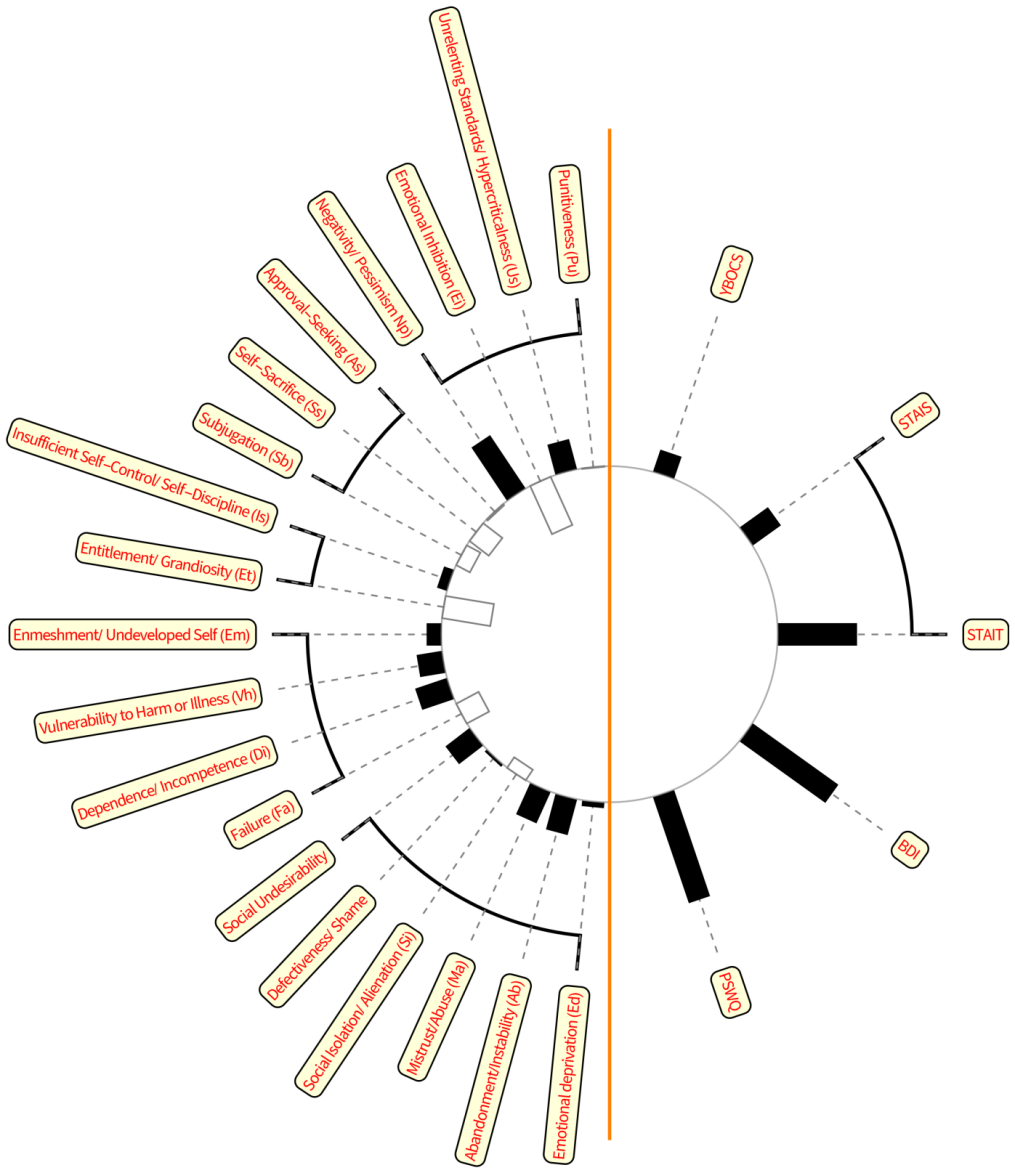
Canonical correlation. Early maladaptive schemas are on the left side, while the symptomatic consequences (anxiety, depression, worry, compulsions) are on the right side. The black columns strengthen the relationship, the white ones indicate the weakening factors.

CCA will find linear combinations of context1 and of context2 variables in such a way that the correlation between the two linear combinations is maximized. The first (most important) unstandardized canonical-correlation coefficients are shown in a helio-map (**Figure 2**); the higher the column the higher the actual value, and the positive values are denoted by solid black. The first canonical correlation is 0.804 (sig < 0.001, F = 2.008, df1 = 95, df2 = 267.352, calculated by SPSS 29.0).

From **Figure 2** we may conclude that the two groups of variables (context1 and context2) correlate highly significantly, and these relationships are dominated by the highest values.

## Discussion

In our study we examined the effect of the presence and severity of early maladaptive schemas on OCD symptoms and on comorbid anxiety and depressive symptoms in a Hungarian sample. We identified five early maladaptive schemas which have a direct effect on the manifestation of obsessive-compulsive symptoms: Mistrust-Abuse, Defectiveness/Shame, Dependence/Incompetence, Entitlement/Grandiosity and Insufficient Self-Control/Self-Discipline. In the case of the Entitlement/Grandiosity schema the effect is reversed. The five identified schemas can be organized into three schema domains: Disconnection and Rejection, Impaired Autonomy and Performance and Impaired Limits.

These schema domains can help the understanding of the pathomechanism of OCD. Our results indicate that problems with attachment stability and security play a significant role in the development of obsessive-compulsive disorder. It seems very likely that OCD patients did not experience attachment security and stability; moreover, they might lack the experience of sharing emotions and receiving valid reflections. The unfavorable early attachment experiences resulted in imperfect, inadequate, or unacceptable self-representations, dominating their self-image. Their basic experiences might also be inhibited and limited fulfilment of their autonomy needs, the feeling of incompetence in relation to their own decisions, and the resulting constant need to seek external confirmation. Additional problems might be the insufficiency of setting up and maintaining boundaries, inadequate regulation and expression of their feelings and impulses, and the active presence of omnipotent fantasies.

In our study, based on the severity of the early maladaptive schemas, we were able to identify three significantly different groups. Among these groups there are significant differences, not only in the appearance and severity of compulsive symptoms, but also in the presence of anxiety and depressive symptoms.

However, contrary to our expectations, the severity of the early maladaptive schemas did not show a linear relationship with the examined symptom variables. According to our results, compulsive, anxiety and depressive symptoms show a stronger correlation with the *number*of early maladaptive schemas even when these are less severe. It seems that the more early maladaptive schemas a person has, the more of his/her basic needs remain unfulfilled, and the more areas of his/her life feature consequential undesirable effects. On the other hand, in the case of fewer maladaptive schemas (even if they are more severe), the person is presumably able to function adaptively in more areas or life situations.

The second goal of the study was to examine the presence and severity of comorbid anxiety and depressive symptoms in OCD patients in the light of early maladaptive schemas.

Our results indicate that OCD symptoms show a significant correlation with anxiety and depressive symptoms. The strongest relationship was found in the case of trait anxiety and OCD.

Using canonical correlation we intended to examine the relationship among early maladaptive schemas, OCD symptoms, anxiety and depressive symptoms from a new perspective.

The results highlight that OCD is only one consequence of early maladaptive schemas and not the most serious one.

In other words, our data indicate that the appearance of compulsive symptoms is only one, and not even the most significant consequence of personality damage.

Based on the results of our study, it can be suggested that obsessive-compulsive disorder is a psychiatric disease that can be described with a complex pathomechanism, where several triggering factors can be identified. Compulsive patients can be classified into subgroups with different symptoms and different patterns of personality impairment. One group of patients is characterized by a severe range of compulsive symptoms in addition to the functional presence of intact personality parts, while another group is characterized by the presence of few compulsive symptoms (which may cause severe subjective discomfort for the patient) and numerous personality dysfunctions. Thus, a well-defined set of maladaptive schemas can even be predictive of the development/severity of compulsive symptoms.

The group of obsessive-compulsive patients with different symptoms and functional characteristics also provides an opportunity for the further development of different treatment and psychotherapy treatment techniques. It is necessary to use different psychotherapeutic techniques to treat patients belonging to different subgroups.

The results of our study suggest that obsessive-compulsive disorder can be divided into several subgroups, which can be separated in terms of symptom severity, comorbid psychiatric symptoms, and personality impairment patterns, and that the relationship between OCD symptom severity and personality impairment is not directly proportional.

### Strengths and limitations

To the best of our knowledge, our study was the first to examine early maladaptive schemas and schema domains specific to obsessive-compulsive disorder in a Hungarian, clinical sample. In the analysis of the schemes we have chosen a new methodology, due to the inconsistencies found in the international literature. Our novel result is the comparison of the presence and severity of early maladaptive schemas and other psychiatric symptoms. At the same time, further studies are necessary to determine the additional characteristics of the compulsive patient subgroups identified in our study. Further investigation is required to determine the symptomatic, endophenotype and biological markers of the subgroups.

## Data availability statement

The original contributions presented in the study are included in the article/supplementary material. Further inquiries can be directed to the corresponding author.

## Ethics statement

The studies involving humans were approved by Medical Research Council Budapest, Hungary. The studies were conducted in accordance with the local legislation and institutional requirements. The participants provided their written informed consent to participate in this study.

## Author contributions

KC: Writing – original draft. ÁM: Writing – review & editing. JM: Writing – original draft.

## References

[1] Poyurovsky M Koran L. Obsessive-compulsive disorder with schizotypy vs. schizophrenia with OCD: diagnostic dilemmas and therapeutic implications. J Psychiatr Res. (2005) 39:399–408. doi: 10.1016/j.jpsychires.2004.09.004 15804390

[2] Atmaca M. Treatment-refractory obsessive compulsive disorder. Prog Neuropsychopharmacol Biol Psychiatry. (2016) 70:127–33. doi: 10.1016/j.pnpbp.2015.12.004 26683174

[3] Lai Y Wang T Zhang C Lin G Voon V Chang J. Effectiveness and safety of neuroablation for severe and treatment-resistant obsessive–compulsive disorder: a systematic review and meta-analysis. J Psychiatry Neurosci. (2020) 45(5):356–69. doi: 10.1503/jpn.190079 PMC7850151 32549057

[4] Carmi L Brakoulias V Arush OB Cohen H Zohar J. A prospective clinical cohort-based study of the prevalence of OCD, obsessive compulsive and related disorders, and tics in families of patients with OCD. BMC Psychiatry. (2022) 22(1):190. doi: 10.1186/s12888-022-03807-4 35300642 PMC8932237

[5] Fullana MA Vilagut G Rojas-Farrerea S Mataix-Cols D de Graaf R Demyttenaere K. Obsessive-compulsive symptom dimensions in the general population: results from an epidemiological study in six European countries. J Affect Disord. (2010) 124(3):291–9. doi: 10.1016/j.jad.2009.11.020 20022382

[6] Strom NI Burton CL Iyegbe C Silzer T Antonyan L Pool R. Genome-Wide Association Study of Obsessive-Compulsive Symptoms including 33,943 individuals from the general population. Mol Psychiatry. (2024), 1–10. 38548983 10.1038/s41380-024-02489-6 PMC11420085

[7] American Psychiatric Association (APA). Practice guideline for obsessive-compulsive disorder (2007). Available online at: https://psychiatry.online.org/guidelines.

[8] The National Institute for Health and Care Excellence (NICE). Obsessive-compulsive disorder and body dysmorphic disorder: treatment clinical guideline, (2005) (2005). Available online at: https://www.nice.org.uk/guidance/CG31/chapter/1-Guidance#steps-35-treatment-options-for-people-with-ocd-or-bdd. 39480980

[9] Franklin M Abramowitz J Kozak M Levitt J Foa E. Effectiveness of exposure and ritual prevention for obsessive-compulsive disorder: Randomized compared with nonrandomized samples. J Consulting Clin Psychol 68. (2000) 4:594–602. doi: 10.1037//0022-006X.68.4.594 10965635

[10] Loerinc AG Meuret AE Twohig MP Rosenfield D Bluett EJ Craske MG. Response rates for CBT for anxiety disorders: Need for standardized criteria. Clin Psychol Rev. (2015) 42:72–82. doi: 10.1016/j.cpr.2015.08.004 26319194

[11] Csigó K. Obsessive position: the new psychoanalytic approach of obsessive-compulsive disorder. Curr Psychol. (2023) 42(7):5407–14

[12] Sookman D Pinard G Beauchemin N. Multidimensional schematic restructuring treatment for obsessions: theory and practice. J Cogn Psychother. (1994) 8:175–94. doi: 10.1891/0889-8391.8.3.175

[13] Young JE Klosko JS Weishaar M. Schema therapy: A practitioner’s guide. New York: Guilford Press (2003).

[14] Kessler RC Berglund P Demler O Jin R Merikangas KR Walters EE. Lifetime prevalence and age-of-onset distributions of DSM-IV disorders in the national comorbidity survey replication. Arch Gen Psychiatry. (2005) 62(6):593–602. doi: 10.1001/archpsyc.62.6.593 15939837

[15] Atalay H Karahan D Caliskan M. Early maladaptive schemas activated in patients with obsessive-compulsive disorder: a cross sectional study. Int J Psychiatry Clin Pract. (2008) 12:268–79. 10.1080/13651500802095004 24937713

[16] Kwak K Lee S. A comparative study of early maladaptive schemas in obsessive–compulsive disorder and panic disorder. Psychiatry Res. (2015) 230:757–62. doi: 10.1016/j.psychres.2015.11.015 26599390

[17] Tenore K Mancini F Basile B. Schemas, modes and coping strategies in obsessive-compulsive like symptoms. Clin Neuropsychiatry. (2018) 15:384–92.

[18] Kim J Lee S Lee S. ): Relationship between early maladaptive schemas and symptom dimensions in patients with obsessive-compulsive disorder. Psychiatry Res. (2014) 215:1. doi: 10.1016/j.psychres.2013.07.036 23962740

[19] Kizilagac F Cerit C. Assessment of early maladaptive schemas in patients with obsessive-compulsive disorder. J Psychiatry Neurological Sci. (2019) 132:14–22. doi: 10.14744/DAJPNS.2019.00003

[20] Lochnrer C Seedat C Toit DU. P: Obsessive-compulsive disorder and trichotillomania: a phenomenological comparison. al.BMC Psychiatry. (2005) 5:2. doi: 10.1186/1471-244X-5-2 PMC546013 15649315

[21] Khosravani V Bastan F Ardestani M Ardekani R. Early maladaptive schemas and suicidal risk in an Iranian sample of patients with obsessive-compulsive disorder. Psychiatry Res. (2017) 255:441–8. doi: 10.1016/j.psychres.2017.06.080 28686949

[22] Wilhelm S Berman NC Keshaviah A Schwartz RA Steketee G. Mechanisms of changes in cognitive therapy for obsessive-compulsive disorder: role of maladaptive beliefs and schemas. Behav Res Ther. (2015) 65:5–10. doi: 10.1016/j.brat.2014.12.006 25544403 PMC4313532

[23] Yoosefi A RajziEsfahani S Pourshabaz A Dolatshahee B Assadi A Maleki F. Early maladaptive schemas in obsessive-compulsive-disorders and anxiety disorders Global. J Health Sci. (2016) 8(10):167–77. doi: 10.5539/gjhs.v8n10p167 27302430

[24] Haaland AT Vogel PA Launes G Haaland VØ Hansen B Solem S. The role of early maladaptive schemas in predicting exposure and response prevention outcome for obsessive-compulsive disorder. Behav Res Ther 40. (2011) 11(11):781–8. doi: 10.1016/j.brat.2011.08.007 21920500

[25] Gross E Stelzer N Jacob G. Treating OCD with the schema mode model. In: The wiley-blackwell handbook of schema therapy. John Wilay and Sons, Oxford (2012). p. 173–84.

[26] Jaeger T Moulding R Yang YH David J Knight T Norberg MM. A systematic review of obsessive-compulsive disorder and self: Self-esteem, feared self, self-ambivalence, egodystonicity, early maladaptive schemas, and self concealment. J Obsessive-Compulsive Related Disord. (2021) 31:100665. doi: 10.1016/j.jocrd.2021.100665

[27] Peeters N van Passel B Krans J. The effectiveness of schema therapy for patients with anxiety disorders, OCD, or PTSD: A systematic review and research agenda. Br J Clin Psychol. (2022) 61(3):579–97. doi: 10.1111/bjc.12324 PMC9544733 34296767

[28] American Psychiatric Association. Diagnostic and statistical manual of mental disorders: DSM-5. 5th ed. Washington: American Psychiatric Association (2013). doi: 10.1176/appi.books.9780890425596

[29] Goodman WK Price L Rasmussen S. The Yale-Brown Obsessive Compulsive Scale (Y-BOCS): Past decelopment, use, and reliability. Arch Gen Psychiatry. (1989) 46:1006–16. doi: 10.1001/archpsyc.1989.01810110048007 2684084

[30] Spielberger CD Gorsuch RL Lushene RE. Manual for the state-trait anxiety inventory. Palo Alto, California: Consulting Psychologist Press (1970).

[31] Sipos K Sipos M. The development and validation of the Hungarian form of the STAI. In: Speilberger CD Diaz Guerro, editors. Cross-cultural anxiety 2. Hemisphere Publishing Corporation, Washington-London (1978). p. 51–61.

[32] At B. An inventory for measuring depression. Arch Gen Psychiatry. (1961) 4:53–63. doi: 10.1001/archpsyc.1961.01710120031004 13688369

[33] Kopp M Foris. N. A szorongás kognitív viselkedésterápiája. Budapest: Végeken sorozat (1993).

[34] Meyer TJ Miller ML Metzger RL Borkovec TD. Development and validation of the penn state worry questionnaire. Behav Res Ther. (1990) 28:487–95. doi: 10.1016/0005-7967(90)90135-6 2076086

[35] Pajkossy P Simor P Szendi I Racsmány M. Hungarian validation of the penn state worry questionnaire (PSWQ). Eur J Psychol Assess.(2014).

[36] Unoka Z Rózsa S Fábián Á Mervó B Simon L. A Young-féle Séma Kérdőív: A korai maladaptív sémák jelenlétés mérő eszköz pszichometriai jellemzőinek vizsgálata. Psychiatria Hungarica. (2004) 19(3):235–43.

